# Kinetics of Photocatalyzed Reactions: Five Lessons Learned

**DOI:** 10.3389/fchem.2018.00378

**Published:** 2018-08-31

**Authors:** David F. Ollis

**Affiliations:** Department of Chemical and Biomolecular Engineering, North Carolina State University, Raleigh, NC, United States

**Keywords:** photocatalysis, kinetic model, Langmuir-Hinshelwood, pseudo-steady state, self-cleaning surfaces, initial rate, reactant size distribution

## Abstract

Elucidation of kinetics of photocatalyzed chemical mechanisms occurring at interfaces (gas-solid, liquid-solid) has been challenging. We summarize here five lessons learned over five decades.

1. An assumed reaction network leads to a single kinetic model, but a common model, the Langmuir–Hinshelwood rate equation, r = k_cat_ K C/ [1 +KC], arises from multiple mechanisms, hence models alone do not reveal unique mechanisms.

2. The Langmuir–Hinshelwood model parameter k_cat_ represents the slow step at a catalyst surface, and in thermal catalysis, depends upon the reactant structure. However, early photocatalysis work with light chlorinated hydrocarbons in aqueous solutions showed a single k_cat_ value, independent of reactant structure.

3. The dependence of the Langmuir-Hinshelwood parameters, k_cat_ and K, upon intensity indicates that a pseudo-steady state approach is more fundamental than the presumed equilibrated adsorption of the LH model.

4. Dyes and phenols are commonly studied, and claimed as first order reactions, despite often exhibiting rate constants which diminish with increasing contaminant concentration. We show that such studies are the result of intrinsic zero order data plotted on a semilog graph, and involve zero order rate limitation by reactant saturation, electron transfer to O_2_, oxygen mass transfer, or light supply.

5. The apparent kinetics for contaminant removal from photocatalytic self-cleaning surfaces depends upon multiple circumstances, including the geometry of reactant deposit, catalyst porosity, and reactant light absorption. A single decision table suffices to indicate the apparent reaction order, n, to assume when fitting photocatalytic kinetic data from self-cleaning surfaces to a power law rate form, rate = k C^n^.

## Introduction

Catalysts, by definition, allow for an increased reaction rate without themselves undergoing any permanent change. Kinetic mechanisms and corresponding reaction rate models are central to characterization and comparisons of catalysts. We summarize here the evolution and refinement of kinetic analyses of photocatalyzed reactions which demonstrate the care and consistency required to obtain and interpret kinetic data. Five examples are discussed which illustrate central issues in photocatalysis.

## Lesson 1: Mechanisms lead to rate forms, but rate forms do not imply unique mechanisms

It is established that photocatalysts produce active species, including OH radicals and h+ holes in the semiconductor (Turchi and Ollis, 1990). The majority of photocatalyzed oxidations in water involve OH radicals, so we considered these only for the present discussion. We imagined four mechanisms by which OH radicals participate in oxidation of reactant, C:

Langmuir–Hinshelwood biomolecular: C(ads) + OH(ads) = > products

Eley–Rideal biomolecular: C(ads) + OH (sol'n) = > products

Eley–Rideal biomolecular: C(sol'n) + OH(ads) = > products

Solution phase oxidation: C(sol'n) + OH (sol'n) = > products.

A detailed analysis (Turchi and Ollis, 1990) indicated that kinetic rate forms resulting from each of these assumed mechanisms yields some version of the Langmuir–Hinshelwood rate form:

(1) $$\begin{array}{l} \text{rate}={\text{k}}_{\text{cat}}\text{KC}/\left[1+\text{KC}\right] \end{array}$$

The conclusion is that in photocatalysis, as in many other areas of kinetics, a given mechanism leads to a unique rate form, but the converse is not true. Thus, the true photocatalyzed mechanism must be established by demonstrations beyond showing merely a satisfactory fit to Equation (1).

We note that other primary oxidizers are discussed as well, including OH radical production from surface ${\text{O}}_{2}^{-}$ lattice oxygen (Montoya et al. (2014), and ${\text{CO}}_{3}^{-}$ radicals in carbonate containing solutions (Xiong et al., 2016). These circumstances both involve photoproduced active centers, the concentrations of which would be intensity dependent, and would again involve a fast reaction step for the active forms, and thus again displace any upstream equilibria, leading again to a required usage of a pseudo-steady state analysis.

## Lesson 2: The photocatalytic oxidation rate constant, k_cat_, is a property of the photocatalyst, and does not depend appreciably upon reactant structure

The classical test for the LH rate form is to plot reciprocal rate vs. reciprocal reactant concentration:

(2) $$\begin{array}{l} 1/\text{rate}=1/{\text{k}}_{\text{cat}}+1/\left[{\text{k}}_{\text{cat}}\text{KC}\right] \end{array}$$

In thermal heterogeneous catalysis, both the strength of adsorption and the magnitude of the rate constant usually vary with reactant structure.

**Figure 1:**
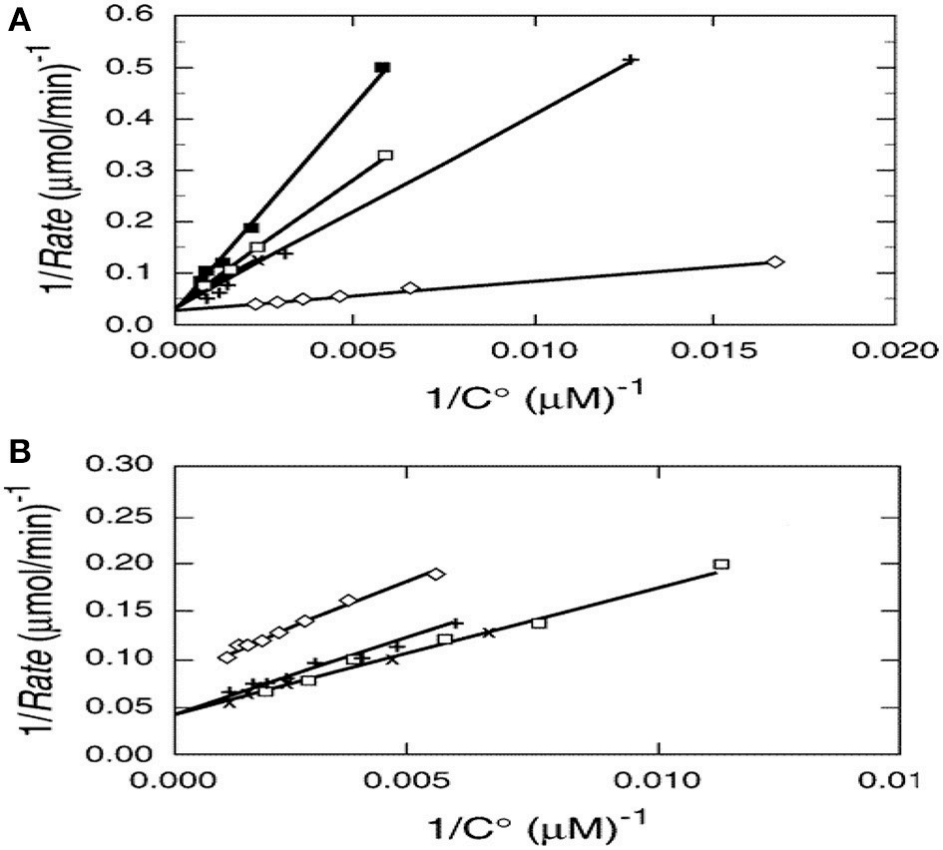
**(A)**Reciprocal initial rate vs. reciprocal initial concentration for (□) chloroform, (x) dicloromethane, (♢) perchloroethylene (PCE), (x) chloroacetic acid, and (+) dichloroacetic acid. **(B)** Reciprocal initial rate vs. reciprocal initial concentration for (□) tribromomethane, (x) dibromomethane, (♢) 1,2 dibromoethane, (+) 1,1-dibromooethane. Catalyst; 1.0 g/L Fisher Chemical TiO2, Lot 773688. Excepting 1,2 dibromomethane, the y-axis intercepts, 1/k_cat_, appear to be identical (Turchi and Ollis, 1990; reprinted by permission of Elsevier).

Photocatalysis provides an interesting exception as shown by kinetic results from multiple papers examining the photocatalyzed destruction of chlorinated and brominated light hydrocarbons. Reciprocal rate vs. reciprocal concentration data plots for photocatalyzed oxidative destruction of these reactants show a common intercept (Turchi and Ollis, 1990) as seen in Figures 1A,B, thus indicating identical values of k_cat_ for all reactants shown. In other words, k_cat_ is essentially independent of reactant structure. If k_cat_ is the product of an intrinsic rate constant and OH concentration (surface or solution), then the product k_cat_ OH is a fundamental measure of photocatalyst activity, and clearly in turn depends on intensity, as OH derives from reactions involving surface species h+ and e– which in turn are produced via photon absorption in the semiconductor.

This intriguing result reflects the better known conclusion from gas phase kinetics: Excepting fully saturated hydrocarbons possessing only methyl groups, most other hydrocarbons exhibit gas phase second order rate constants for (OH + hydrocarbon) which are similar, of the order of 10^−11^ cm^3^ molecule^−1^ s^−1^ (Atkinson et al., 2006), and thus essentially independent of reactant structure.

## Lesson 3: Photocatalysis kinetics require a pseudo-steady state analysis because the equilibrium adsorption model is invalid

The earliest photocatalysis papers routinely utilized Langmuir-Hinshelwood rate forms for one simple reason: Photocatalysis is a subfield of heterogeneous catalysis, and the latter field has historically been dominated by this analytic function,

(3) $$\begin{array}{l} \text{rate}={\text{k}}_{\text{cat}}{\text{K}}_{\text{ads}}\text{C}/\left[1+{\text{K}}_{\text{ads}}\text{C}\right] \end{array}$$

where k_cat_ is a fundamental representation of catalyst activity, and K_ads_ is, historically, the adsorption equilibrium constant of the Langmuir isotherm,

(4) $$\begin{array}{l} {\text{K}}_{\text{ads}}={\text{k}}_{1}/{\text{k}}_{-1} \end{array}$$

where k_1_ and k_−1_ are reactant adsorption and desorption constants, respectively.

In photocatalysis, k_cat_ is the product of a rate constant, k′, and some active specie such as OH radicals or semiconductor hole concentration, h+. Since these active species are the presumed result of photoactivation, their concentrations, and thus the catalytic rate constant, k_cat_, are expected to be a function of light intensity, I. In contrast, the traditional Langmuir-Hinshelwood approach assuming adsorption/desorption equilibrium contains rate constants k_1_ and k_−1_ which are not assumed to depend on intensity. The experimental observation that not only k_cat_, but also K_ads_, depend upon intensity (Emeline et al., 2000; Ollis, 2005; Mills et al., 2015) indicated failure of the equilibrated adsorption assumption.

This situation has been rectified by invoking a pseudo-steady state (PSS) assumption, commonly used in other kinetic analyses involving highly unstable intermediates such as free radicals. This analysis allows k_cat_ to have any value relative to k_−1_. In particular, the PSS analysis leads to an apparent adsorption constant, K_ads_, which depends upon k_cat_, and thus varies with intensity (Ollis, 2005):

(5) $$\begin{array}{l} {\text{K}}_{\text{ads}}\left(\text{apparent}\right)={\text{k}}_{1}/\left({\text{k}}_{-1}+{\text{k}}_{\text{cat}}\right) \end{array}$$

Because all photocatalyzed aqueous phase oxidations carried out in the presence of water and molecular oxygen produce the same active species, OH and h+, we may expect that all such conversions require PSS rather than Langmuir-Hinshelwood analyses.

Mills et al. (2015) have termed this pseudo steady state approach the “disrupted adsorption kinetic model” and analyzed it along with other pseudo-steady state models for photocatalyzed reactions, paying particular attention to the most promising, those of Gerischer (1995); Emeline et al. (2000), Valencia et al. (2011), and Montoya et al. (2014). They concluded that “The best of those tested, in terms of overall fit, simplicity, usefulness, and versatility is the disrupted adsorption kinetic model proposed by Ollis.”

## Lesson 4: Only initial rate analysis reveals intrinsic reaction order, n, whereas temporal data may yield kinetic disguises

Oxidation of organic reactants has been one of the most investigated aspects of research in photocatalysis. Two of the most frequently studied reactant classes are phenols and dyes, for which Web of Science (5/20/2018) indicates that 5,363 and 19,129 papers, respectively have been published to date.

A number of these reports find that semilog plots of reactant concentration, C(t), vs. time yield linear forms, but that the slope of such plots, and thus the apparent first order rate constant, k_cat_, decrease with increased initial concentration, C_o_ These results indicate that the presumed rate constant (slope) of such plots is not, in fact, a constant, and thus that the assumption of first order mechanism is incorrect, as we demonstrate for phenol.

**Figure 2:**
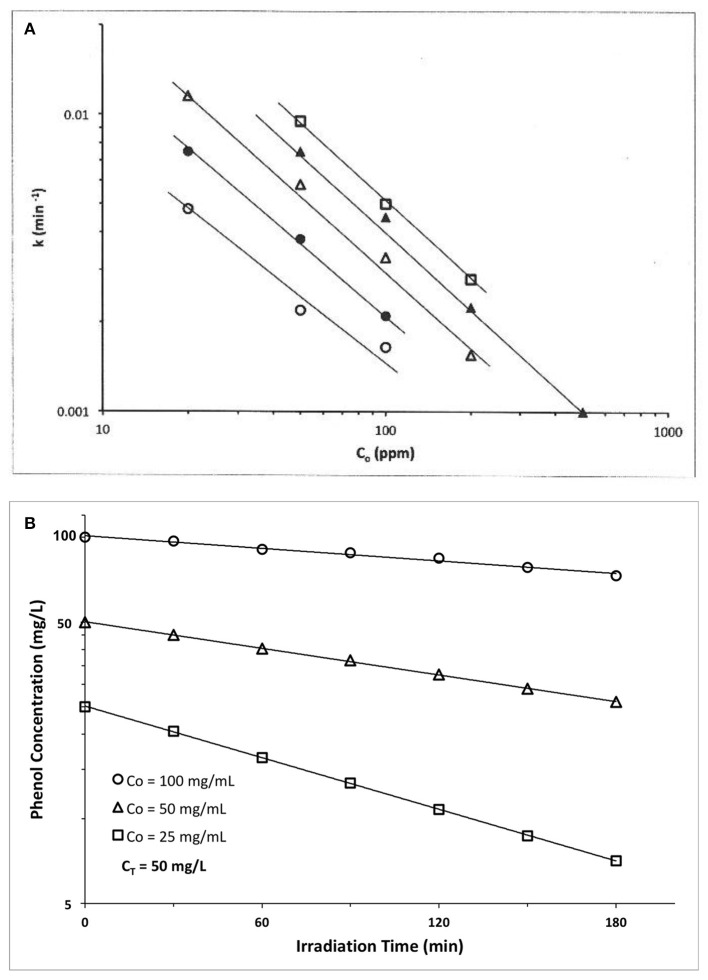
**(A)** Photocatalyzed oxidation of phenol by illuminated ZnO. Semilog plots of apparent first order rate constants vs. initial solution concentration. All plots exhibit slopes of approximately −1, confirming our claim that these data actually represent a zero order reaction, because the y-x product of apparent first order rate constant(y) times the initial concentration(x) is a constant, i.e., (yx) = k_1_ C_o_ = k_o_ = zero order rate ((Kawaguchi, 1983); Reprinted with permission). **(B)** Photocatalyzed oxidation of phenol by illuminated TiO2(anatase). The product of apparent first order rate constant (slope) times the initial concentration, Co, is a constant, of average value 0.194 mg phenol/l-min (Table 1), thus indicating presence of a zero order reaction (Kawaguchi, 1984).

An early example is provided by Kawaguchi (1983) who explored phenol photocatalyzed oxidation by ZnO using a high pressure Hg lamp. At each of five different ZnO photocatalyst concentrations (C_z_), a ln-ln plot of apparent first order rate constant vs. initial phenol concentration, C_o_, yielded a straight line (Figure 2A). We claim these results represent a disguised zero order process. We first establish zero order rate, then later explain the apparent first order temporal result.

At each catalyst concentration in Figure 2A, the doubly logarithmic plot of apparent first order rate constant [evaluated from ln C(t) vs. t plots] vs. initial concentration, Co, is linear with negative slopes of −0.85, −0.91, −0.1.04, −0.97, and −0.95, respectively, for five different catalyst concentrations. Since we claim here that k_o_ = k_1_(apparent) C_o_, then ln k_1_ = ln k_o_ – ln C_o_, and a graph of ln k_1_ vs. ln C_o_ should have a slope of −1.0, as verified by the experimental results of Figure 2B and the slopes calculated therefrom. Thus, this photocatalyzed reaction exhibits a zero order behavior, because the product of apparent first order rate constant, k_1_, times initial phenol concentration, C_o_, is a constant, k_o_. The zero order constant, k_o_, is, however, dependent on photocatalyst concentration as Figure 2A indicates.

**Table 1:** ZnO photocatalyzed oxidation of phenol Kawaguchi, 1984.

**Data**	**Data**	**Calculated initial rate**
C_o_(mg/l)	k_1_(apparent) (min^−1^)	r_o_(mg/l-min) = k_1_ C_o_ = k_o_
100	1.94 × 10^−3^	0.194
50	3.8 × 10^−3^	0.190
25	7.9 × 10^−3^	0.198

A second example is also provided by Kawaguchi (1984) for the TiO_2_ (anatase) photocatalyzed oxidation of phenol (Figure 2B). The initial rate of reaction we recover from the Figure 2B results by multiplying each slope (k_1_, apparent first order rate constant) × its initial phenol concentration, C_o_. If the rate is truly zero order, then the initial rate, r_o_ = k_o_ = k_1_ C_o._ = should again be constant, so we calculate r_o_ from the product k_1_ C_o_ to recover the experimental initial rate results which appear in the Table 1 below.

Thus, all three data sets in Figure 2B are represented by a zero order average rate of magnitude k_o_ = 0.194 mg/l-min !

**Figure 3:**
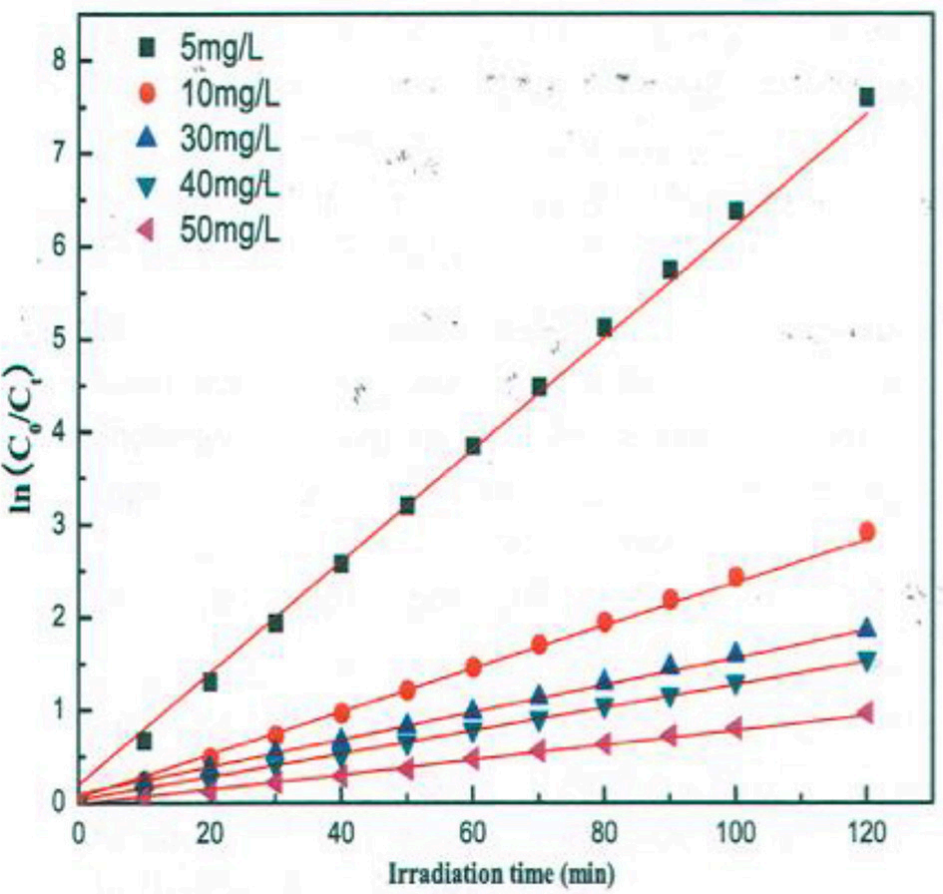
Kinetics of methyl orange degradation catalyzed by ZnO nanoparticles (Chen et al., 2011; Reproduced by permission).

**Figure 4:**
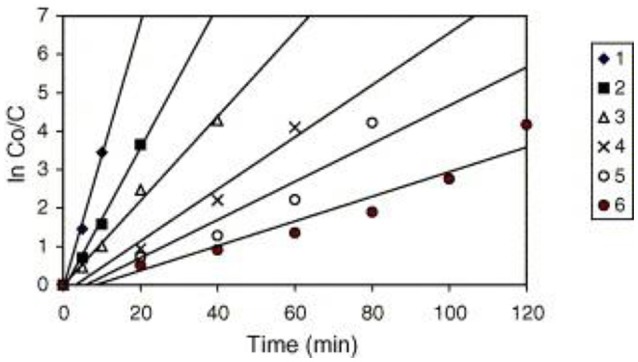
Kinetics of RY14 decolorization by UV/TiO2-P25 for different initial dye concentrations. TiO2-P25 = 4 g/l, pH 5.5 ± 0.1. [1] = 1 × 10^−4^ mol/l, [2] = 2 × 10^−4^ mol/l, [3] = 3 × 10^−4^ mol/l, [4] = 5 × 10^−4^ mol/l, [5] = 7 × 10^−4^ mol/l, and [6] = 9 × 10^−4^ mol/l ((Muruganandham and Swaminathan, 2006); Reprinted by permission).

**Figure 5:**
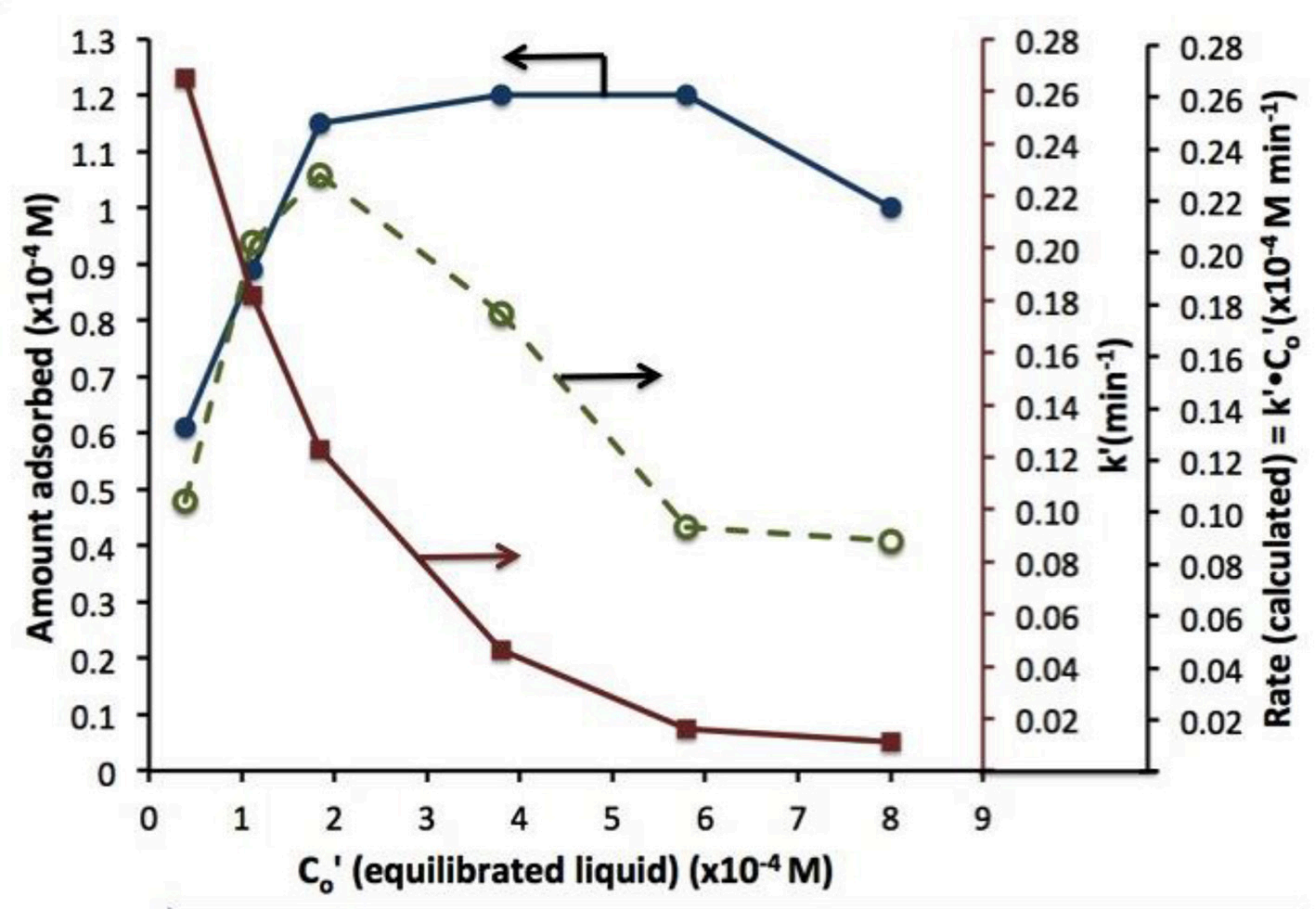
Dye absorbed (left axis), rate constant k'(closed circles), and rate (calculated) vs. equilibrated liquid phase dye concentration (open circles). Beyond 2 10^−4^ M dye, the coverage is complete; eventual rate decline at higher Co is likely due to photon absorption by dye.

A similar behavior of first order rate constants varying with C_o_ is periodically reported for photocatalyzed oxidation of organics dyes (e.g., Chen et al., 2011; Figure 3) and (Muruganandham and Swaminathan, 2006; Figure 4). Prabha and Lathasree (2014) also found this behavior; Prabha's thesis contains dye date showing initial loading of dye, and resultant bulk phase equilibrated dye concentration, from which the initial amount of dye adsorbed can be calculated. This calculation (Figure 5) shows that the photocatalyst surface was saturated in dye over nearly the entire concentration range studied, even though an apparent first order behavior for each C_o_ value was reported. Such saturation implies a zero order rate of reaction as we have argued above.

This dye situation is more complicated that the phenol example above, as photolysis, photocatalysis, and photosensitization may all play a role, depending on the dye, wavelength, and photocatalyst used. These cases are analyzed in a future paper, but the “take away” message is already clear: variable first order rate “constants” strongly suggest a kinetic disguise of e.g., a zero order process, be it due to reactant saturated surface, oxygen supply limitation, or light limitation.

It is curious that authors of such studies have limited their kinetic analyses to semilog plots to determine reaction order, rather than also exploring the zero order possibility. The existence of a zero order rate of reaction may arise from multiple circumstances:

(a) The surface is saturated in organic reactant, e.g., phenol.

(b) The reaction rate is limited by some other participant, e.g., light supply or oxygen mass transfer (Ollis et al., 2015). In each case, the constant, but limiting, supply of light or oxygen would lead to a zero order rate of reaction.

The observed linearity of semilog plots of C(t) vs. time shown in Figure 2A and Table 1 then requires an alternate explanation, if the initial reaction rate is truly of zero order. We consider competition for oxidant between the original reactant and a series of also oxidizable, organic intermediates (Ollis et al., 2015). The fraction of oxidant consumed by the initial dye reactant is given by

(6) $$\begin{array}{l} {\text{f}}_{\text{dye}}={\text{k}}_{\text{dye}}{\text{C}}_{\text{dyc}}/\left[{\text{k}}_{\text{dye}}{\text{C}}_{\text{dye}}+\sum {\text{k}}_{\text{i}}{\text{C}}_{\text{i}}\right] \end{array}$$

where k_dye_, k_i_, are the second order rate constants for reaction of dye and intermediates, i, respectively with the rate limiting oxidant, e.g., OH radical. Since the k_i_s are expected to be similar, the denominator is expected to be relatively constant (the sum term grows as the dye concentration term decreases over time, and the denominator will remain approximately constant). As a result, the reaction rate, proportional to OH × f_dye_, will exhibit an apparent first order behavior over time, even while the initial rate is zero order within the concentration range studied.

## Lesson 5: The apparent order of reaction, N, for photocatalyzed self-cleaning of deposits on (air)-solid(photocatalyst) surfaces may exhibit a value lying between 0 and 2, and depends on five reaction conditions

Two surveys and analyses of literature data for photocatalytic self-cleaning surfaces (Ollis, 2010, 2017) show that when rate data are analyzed by fitting a power law expression,

(7) $$\begin{array}{l} \text{rate}={\text{k}}_{\text{cat}}{\text{C}}^{\text{n}}\left(\text{t}\right) \end{array}$$

the value of *n* found falls in the range 0 < *n* < 2. The apparent value of n revealed for a given set of experiments depends on the answers to five questions:

(i) Is the photocatalyst porous or non-porous ?

(ii) Is the reactant deposited on the outer surface, or distributed within a porous catalyst ?

(iii) Is the photocatalyst optically thick (OD > 2) or thin (OD < 0.3)?

(iv) Is the reactant deposited as a submonolayer or a multilayer ?

(v) Is the reactant present as a continuous film or as discrete deposits? If as discrete deposits, is the deposit size distribution monodisperse, narrow, or broad?

**Figure 6:**
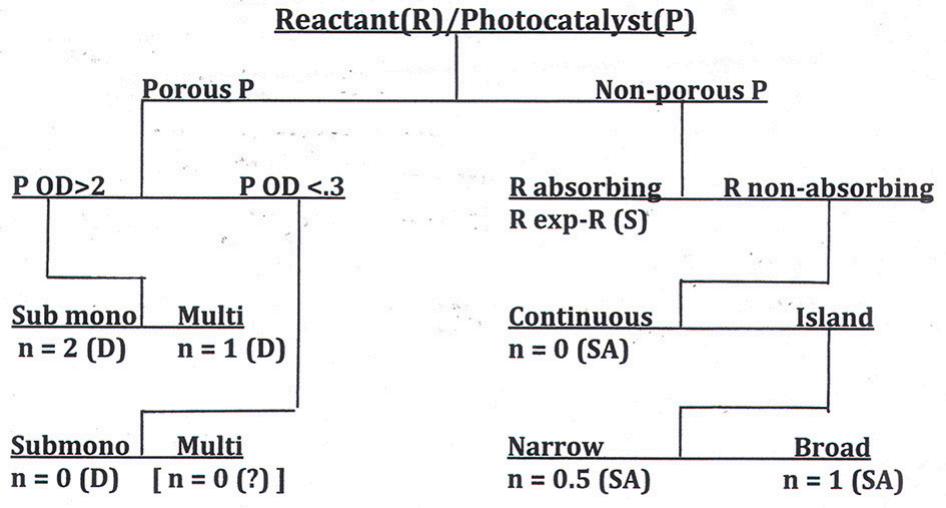
A decision tree for determination of reaction order, n, for the photocatalyzed removal of deposits from surfaces. P, photocatalyst; R, reactant; D, dye; SA, stearic acid; S, soot (Ollis, in press).

We have shown elsewhere (Ollis, in press) that, equipped with the answers to these questions, the reaction order, *n*, can be predicted. The possibilities for catalyst-reactant circumstances are collected in a decision tree diagram, a format commonly used in statistical analyses when the outcome of a question, e.g., “What is the apparent order of reaction, *n*?” depends on the answers to multiple queries, each of which requires only a binary (e.g., yes/no) response. This decision table, which should guide those seeking to measure activity of self-cleaning surfaces, appears in Figure 6.

## Conclusion

Analysis of the kinetics of photocatalyzed reactions was early on assumed to be rather simple, as early data for liquid-solid systems, as well as gas-solid examples, were fitted to Langmuir-Hinshelwood rate forms, with the equilibrium adsorption assumption implicit in these reports. More detailed analyses have revealed a more complex world, in which the five lessons cited above serve to aid current researchers in finding more fundamental, if more complicated, rationales for their observed kinetic behaviors.

## Author contributions

The author confirms being the sole contributor of this work and approved it for publication.

### Conflict of interest statement

The author declares that the research was conducted in the absence of any commercial or financial relationships that could be construed as a potential conflict of interest.
